# Endoscopic Band Ligation of Bleeding Duodenal Varices

**DOI:** 10.7759/cureus.22009

**Published:** 2022-02-08

**Authors:** Monica Dzwonkowski, Umair Iqbal, Seth W Kaufer, Yakub I Khan, Kishore Kumar

**Affiliations:** 1 Internal Medicine, Geisinger Medical Center, Danville, USA; 2 Gastroenterology, Geisinger Medical Center, Danville, USA; 3 Gastroenterology, Geisinger Health System, Wilkes-Barre, USA

**Keywords:** coil embolization, variceal bleeding, portosystemic shunt, endoscopic band ligation, ectopic variceal bleeding, duodenal varices

## Abstract

Ectopic variceal bleeding is an uncommon cause of gastrointestinal bleeding and carries a high mortality. Management depends on provider comfort and resource availability as treatment guidelines are lacking due to the infrequent occurrence of bleeding ectopic varices. We present a case of a middle-aged woman who presented with melena and anemia requiring transfusion. She was diagnosed with cirrhosis, and computed tomography of the abdomen revealed active bleeding at the proximal duodenum. She underwent emergent esophagogastroduodenoscopy, which showed actively bleeding duodenal varices secondary to portosystemic shunt from portal hypertension. Endoscopic hemostasis was achieved with variceal band ligation, a useful modality when alternative methods of emergent variceal management are unavailable. Given the risk of recurrent bleeding, the patient underwent embolization of varices by interventional radiology.

## Introduction

Variceal bleeding is a common cause of upper gastrointestinal bleeding in cirrhotic patients. Although the stomach and esophagus are the most common sites of varices, they can infrequently be found in ectopic locations such as the rectum, appendix, small intestine, colon, and biliary tract [1]. Ectopic variceal bleeding is an uncommon phenomenon, occurring in approximately 2%-5% of variceal bleeding [1]. Duodenal varices account for only 17% of total ectopic variceal bleeding and have an estimated mortality rate of 40%, attributed to increased vascularity of the duodenum and the deep serosal location of duodenal varices [1-3]. Management of bleeding ectopic varices usually requires an endoscopic treatment or radiologic intervention and rarely surgical intervention. We present a case of a middle-aged woman who presented with acute blood loss anemia secondary to large bleeding duodenal varices fed by a portosystemic shunt from her superior mesenteric vein to the gonadal vein.

## Case presentation

A 52-year-old female with a medical history of gastroesophageal reflux disease, hypertension, neuropathy, morbid obesity, and asthma presented with symptoms of dyspnea and melena for four days. She admitted to taking naproxen several times daily. She denied abdominal pain, weight loss, hematemesis, or hematochezia. There was no history of significant alcohol use. Her family history was significant for alcohol-related liver disease.  She had never had a prior esophagogastroduodenoscopy (EGD). She was mildly tachycardic with otherwise normal vitals. She was alert and oriented.

Abdominal examination revealed mild generalized tenderness without guarding or rigidity. Initial laboratory workup showed hemoglobin 7.2 g/dL (ref: 12-15.3 g/dL). Repeat hemoglobin was 5.7 g/dL despite receiving 1 unit of packed red blood cells. Other lab work included INR 1.20 (ref: 0.84-1.14), platelet count 199 K/uL (ref: 140-400 K/uL), AST 46 U/L (ref: 10-35 U/L), ALT 16 U/L (ref: 10-35 U/L), alkaline phosphatase 51 U/L (35-130 U/L), and total bilirubin 0.3 mg/dL (ref: <1.2 mg/dL). BUN on admission was 35 mg/dL (ref:6-20mg/dL) and creatinine was 0.8 mg/dL (ref:0.5-1.1 mg/dL). 

**Figure 1:**
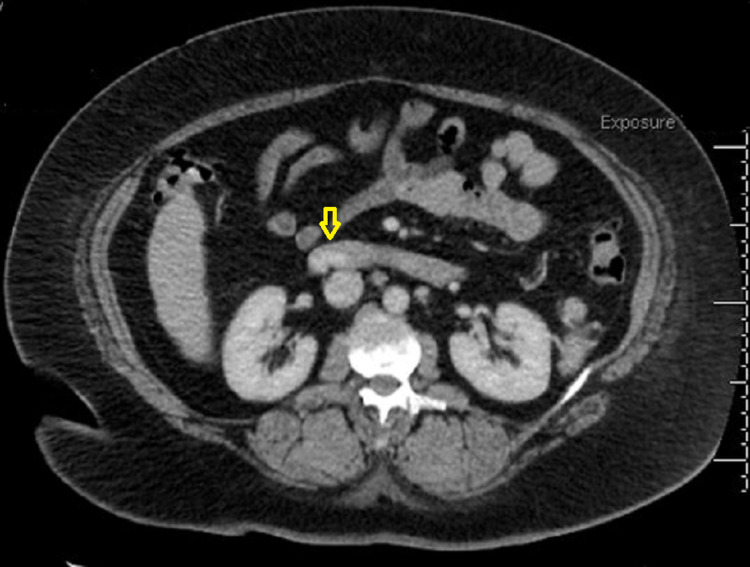
CT scan showing active bleeding in the proximal region of duodenum

Computed-tomography (CT) scan of the abdomen performed in the emergency department identified active bleeding in the proximal duodenum arising from an adjacent branch of the superior mesenteric vein and a nodular contour of the liver concerning cirrhosis (Figure 1). The calculated model for end-stage liver disease (MELD) score was nine, and she was classified as Child-Pugh Class A. The patient was adequately resuscitated with intravenous fluids, octreotide, antibiotics, and proton pump inhibitors.

**Figure 2:**
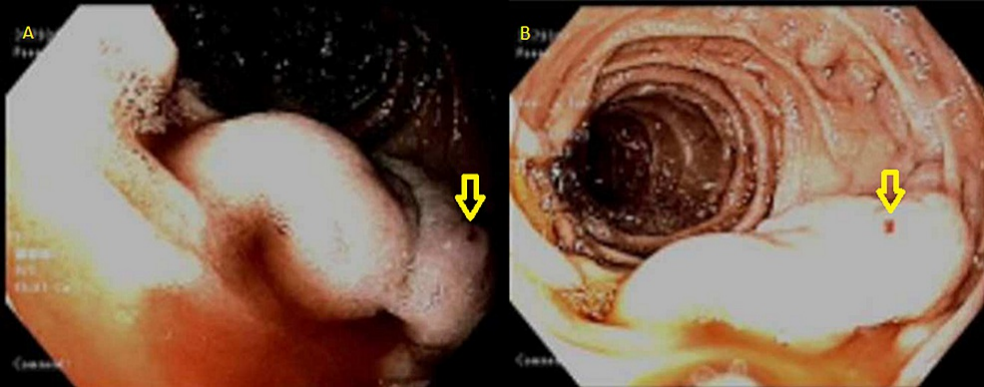
A and B: Endoscopic images showing actively bleeding, large duodenal varix

**Figure 3:**
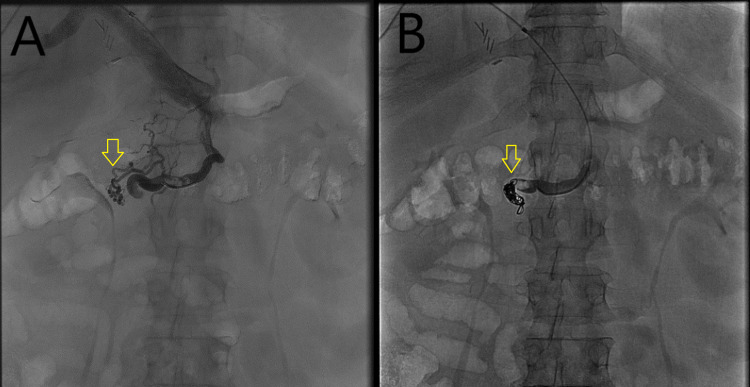
A and B: Fluoroscopic images showing duodenal varices before (3A) and after (3B) coil embolization.

An EGD was performed, and she was noted to have large actively bleeding varices in the second portion of the duodenum (Figure 2A, 2B). Given the unavailability of glue injection or sclerosing agent, the bleeding varices were treated with four rubber bands with adequate hemostasis achieved. Non-bleeding large esophageal varices were also treated with rubber bands during index endoscopy. On further review of her CT abdomen, a shunt was identified between the superior mesenteric vein and gonadal vein, which was supplying the duodenal varices. Subsequent transhepatic embolization of the duodenal varices and coil embolization of the segment of the shunt supplying the varices was performed (Figure 3A, 3B). Repeat testing showed stable hemoglobin without evidence of recurrent bleeding. Liver chemistries were grossly unchanged following the embolization. The patient was discharged home with a follow-up appointment for liver transplant evaluation and further management of cirrhosis. 

## Discussion

The pathophysiology of varices is related to increased portal venous pressure, usually secondary to liver cirrhosis, which leads to portosystemic collateral pathways. Other causes of portal hypertension include a portal or splenic vein occlusion, tumor invasion, or thrombosis [2-4].  The diagnosis of duodenal varices is best accomplished via direct visualization by endoscopy, though a small or distal varix can be misdiagnosed even by an experienced operator. Clinicians should maintain a high index of suspicion for ectopic varices in a patient who continues to show signs of bleeding despite adequate treatment of esophageal varices since bleeding duodenal varices can be devastating [2,3,5]. Additional diagnostic tests can be performed for the diagnosis of ectopic varices, including computed tomography with intravenous contrast, tagged red blood cell scans, mesenteric angiography, and rarely, surgical exploration [4,6].

The endoscopic treatment of duodenal varices is mainly dependent on the preference of the endoscopist and available treatment modalities since there are currently no established standard society guidelines [7]. The American Association for the Study of Liver Diseases guidelines recommends a multidisciplinary approach between hepatologists, endoscopists, surgeons, and interventional radiologists for the management of ectopic varices [6]. The primary goal is to achieve prompt hemostasis and address etiology of the ectopic varix to prevent recurrent bleeding [3]. 

Endoscopic management of duodenal varices, including sclerotherapy with agents such as ethanolamine and polidocanol, ligation, and clips, has taken over as mainstays of treatment. Park et al. successfully treated duodenal varix with the placement of hemoclips [4]. Shiraishi et al. reported successful hemostasis of duodenal varix with endoscopic band ligation alone [8]. In our patient, endoscopic band ligation was performed with the achievement of adequate hemostasis.

House et al. also reported utilizing banding for bleeding duodenal varices. However, due to rebleeding, the patient required placement of a transjugular intrahepatic portosystemic shunt (TIPS) [5]. Similarly, Yohida et al. reported initial treatment with endoscopic banding in a patient with ruptured duodenal varices. The patient later required treatment with injection sclerotherapy with N-butyl-2-cyanoacrylate due to recurrent bleeding [9]. 

Interventional radiological (IR) procedures such as TIPS and balloon-occluded retrograde transvenous obliteration (BRTO) have been successfully used for the treatment of duodenal varices, especially when endoscopic interventions are not successful in controlling bleeding [10]. A study including seven patients evaluated the efficacy of BRTO and showed achievement of hemostasis in all but one patient [11]. IR procedures can also be considered after hemostasis is achieved via endoscopic means to prevent rebleeding, which is what was chosen for our patient as sclerotherapy was not available at the time of admission. 

Surgical treatments such as excision, ligation, and partial duodenectomy proved to have high post-surgical mortality rates and have since fallen out of favor [4]. However, surgery may be necessary in some cases if the duodenal varix is not amenable to endoscopic treatment. Khor et al. reported a rare case of recurrent upper gastrointestinal bleeding requiring multiple EGDs and eventually found to have bleeding duodenal varices in the second and third portion of the duodenum. The patient was taken urgently to the operating room for duodenectomy with good recovery [1].

## Conclusions

In summary, endoscopic band ligation of duodenal varices is an easy, safe, and widely available treatment modality in achieving the initial hemostasis of actively bleeding duodenal varices. There is a high risk of rebleeding, and thus in many cases, treatment requires a multidisciplinary approach for definitive therapy. Interventional radiology procedures such as TIPS, BRTO, and embolization of portosystemic shunts will curtail the risk of re-bleeding, even after successful endoscopic hemostasis is achieved. Though duodenal varices are relatively rare, our case highlights the importance of prompt treatment to prevent devastating consequences.
